# Paraoxonase 1 in Chronic Kidney Failure

**DOI:** 10.1155/2012/726048

**Published:** 2012-03-07

**Authors:** Alejandro Gugliucci, Kazuhiko Kotani, Satoshi Kimura

**Affiliations:** ^1^Glycation, Oxidation and Disease Laboratory, Touro University-California, Vallejo, CA 94592, USA; ^2^Department of Clinical Laboratory Medicine, Jichi Medical University, Tochigi 329-0498, Japan; ^3^Department of Laboratory Medicine and Central Clinical Laboratory, Showa University Northern Yokohama Hospital, Kanagawa 224-8503, Japan

## Abstract

In this review we summarize the findings from the literature and our own laboratory on the decreased PON1 activity in renal failure, the mechanisms proposed and the effect of interventions. In addition to profound alterations in lipoproteins, reduced serum PON1 activity has been clearly established in the past decade and could contribute to accelerated development of atherosclerosis in ESRD and in HD. PON1 lactonase activity is lower in ESRD patients. Hemodialysis partially restores PON1 lactonase and the other activities. PON1 activity recovery after dialysis suggests that uremic toxins may play a mechanistic role in PON1 inactivation. Lower PON1 activity in CRF patients is associated with low thiol concentration, high CRP, and is beneficially enhanced with vitamin C and flavonoids. Changes in HDL subclasses, namely lower HDL_3_ in these patients may also play a role in PON1 lower activity. Future research should focus on: (1) mechanistic studies on causes for low PON1 activity and mass; (2) prospective studies focusing on whether there is an added predictive value in measuring PON1 activity (and PON1 activity in HDL_3_) in this patient population; (3) intervention studies attempting to increase PON1 activity.

## 1. Introduction

The major cause of mortality in patients with end-stage renal disease (ESRD) receiving renal replacement therapy is cardiovascular disease. More than one million of these patients throughout the world are surviving with the assistance of renal replacement therapy [1–8]. More than 800,000 patients receive hemodialysis (HD), the most frequent modality. Survival on HD has improved, although vascular accidents, such as ischemic heart disease and hemorrhagic stroke, remain major problems [2, 7, 8]. All patients with chronic renal failure (CRF) have increased risk for death from cardiovascular disease, especially those undergoing HD [1, 2, 9]. They have numerous metabolic disorders that may hasten the development of plaques, such as insulin resistance, hypertension, and dyslipoproteinemia, along with other ESRD-related risk factors such as the classical calcium and phosphate metabolism disorders and secondary hyperparathyroidism [1–9]. CRF patients frequently have lipoprotein abnormalities such as low high-density lipoprotein (HDL)-cholesterol concentrations, increased remnant particles and hypertriglyceridemia. HDL-cholesterol concentrations are inversely correlated with atherogenic risk [3, 4, 6, 7].

HDL is not only a key player in reverse cholesterol transport but has the ability to protect low-density lipoprotein (LDL) against oxidation, is an anti-inflammatory mediator, protects the endothelium, and modulates coagulation [10–14]. There is mounting evidence that paraoxonase 1 (PON1) could be implicated in several of these processes, as shown in detail elsewhere in this special issue of this journal [15–26]. Human PON1 (aryldialkylphosphatase, EC 3.1.8.1) is an esterase associated with apolipoprotein AI (apoAI) and clusterin (apolipoprotein J) in HDL. PON1 displays paraoxonase and arylesterase activities since it hydrolyzes organophosphate compounds such as paraoxon, and aromatic carboxylic acid esters such as phenylacetate. PON1 possesses peroxidase-like activity that can contribute to its protective effect against lipoprotein oxidation [22, 27]. It also displays homocysteine-thiolactonase activity that may be linked with its antiatherogenic properties [28, 29]. PON1 protects lipids in lipoproteins, macrophages and erythrocytes from oxidation [30–32]. Together with its antioxidative properties, PON1 has added antiatherogenic activities against macrophage foam cell formation: reduction of cholesterol and oxidized lipids influx, inhibition of macrophage cholesterol synthesis, and stimulation of macrophage cholesterol efflux [30–32]. However, the mechanism of PON1's protective action and its endogenous substrate remain elusive. Evidence is accumulating indicating that the lactonizing/lactonase activity of PON1 may be physiologically the most significant. Lactonase activity is exerted on oxidized phospholipids and on homocysteine-thiolactone [33–37] Hyperhomocysteinemia, encompassing also higher concentrations of homocysteine-thiolactone, is common in both ESRD and in patients on dialysis and may be an added risk factor for enhanced atherogenesis.

In the past decade, much progress has been made on PON1 status in patients with renal failure. Several case-control studies have addressed the changes in PON1 activity and mass as well as prevalence of polymorphisms. The effect of therapeutic modalities of intervention on PON1 activity has been explored: hemodialysis versus conservative treatment; hemodialysis and transplant; peritoneal dialysis; different types of dialysis membranes; erythropoietin; vitamin C and quercetin.

**Figure 1:**
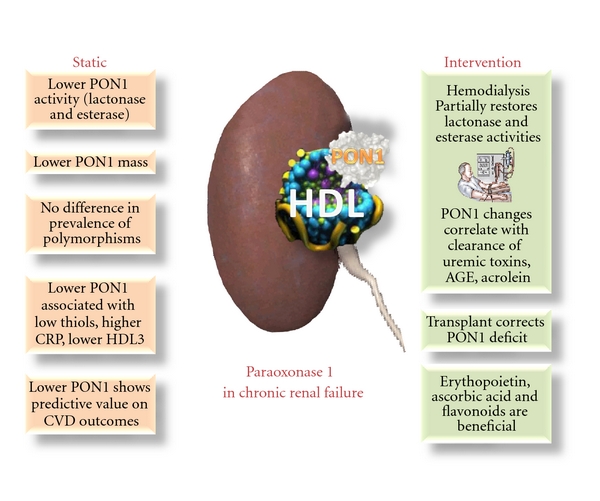
Paraoxonase 1 activity and function are compromised in chronic renal failure patients. This diagram summarizes the main findings discussed in the text as reported during the past decade in the literature.

In this paper, we summarize the findings from the literature and our own laboratory on the decreased PON1 activity in renal failure, the mechanisms proposed, and the effect of interventions. A bird's eye view of the main findings is presented on Figure 1.

## 2. PON1 Activity is Lower in Chronic Renal Failure

In1998, two groups first studied PON1 activity in patients with CRF as compared to control subjects. In one of these studies, 119 hemodialyzed CRF patients, 107 patients with primary hyperlipoproteinemia, and 110 healthy control subjects were recruited and studied. PON1 activity was significantly lower both in hyperlipidemic and renal failure patients as compared with controls [38]. To assess whether the reduction in PON1 activity was due to changes in HDL and Apo-A1 levels, the authors standardized the enzyme activity for HDL and Apo-A1 concentrations. The standardized PON1 activity (paraoxonase/HDL ratio) was also lower in the renal failure patients as compared with hyperlipidemic patients and controls. The phenotypic distribution of paraoxonase was not different in the patient groups [38]. The same year, another group reported a study on 305 patients with CRF, 47 patients with non-ESRD, 22 patients treated with peritoneal dialysis 104 patients treated with hemodialysis, and 132 renal transplant patients [39, 40]. Patients were compared with two groups of aged-matched control subjects (*n* = 195). PON1 activity was lower in patients with renal insufficiency (chronic renal failure; chronic hemodialysis; chronic peritoneal dialysis) than in control subjects. Renal transplantation seems to restore PON1 activity. The same group then reported that PON1 in renal failure patients is better activated by salt than that of control subjects, suggesting qualitative changes in the molecule [39, 40]. These findings have since been confirmed by several groups in multiple studies [41–49].

## 3. PON1 Concentration is Lower in Chronic Renal Failure

The fact that PON1 activity is lower in the sera of CRF patients is well established as stated above. This led to multiple studies addressing the mechanism behind this change. The first issue to clarify is whether the PON1 protein is also low, or it is only a qualitative affect (inhibition or shift in HDL composition). Serum PON1 concentrations were measured in 81 patients undergoing hemodialysis and 103 age-matched healthy subjects using an enzyme immunoassay [50]. PON1 concentration was significantly lower in the patient group than the control group. There were no significant associations between serum PON1 concentrations and the PON1 genetic polymorphisms, 55Leu/Met (L/M), and 192Gln/Arg (Q/R). The concentration adjusted for HDL-cholesterol or apolipoprotein A-I was also lower in the patient group. Several other studies have since confirmed these findings in other series [50–52].

## 4. Hemodialysis versus Continuous Ambulatory Peritoneal Dialysis (CAPD), Conservative Treatment, Transplant

 Several reports are consistent to show that PON1 activity and mass is lower at predialysis in CRF patients under HD or CAPD or other modalities, with no differences between these groups [42, 53]. Transplanted patients display no differences with controls [49].

## 5. PON1 Polymorphisms Prevalence Does Not Differ in Chronic Renal Failure Patients and Control Subjects

 The PON1 genetic polymorphisms of 192 Gln/Arg and 55 Leu/Met in the amino acid sequence are partly involved in the PON1 enzyme activity. Namely, the 192 Gln/Arg QQ homocygotes have much lower PON1 activities when measured with paraoxon and not so significant with other substrates. Therefore, it is important to investigate whether the polymorphisms are associated with CRF, since a shift in the prevalence may provide an explanation for the lower activity found consistently in the aforementioned studies. Several papers have addressed this issue, some employing molecular biology techniques, some using functional phenotyping with 2 substrates, and finally some using combined approaches. In 2000, the two polymorphisms (192 Gln/Arg and 55 Leu/Met) were assessed in 96 patients undergoing hemodialysis and in 136 normal controls [52]. There was no difference in the distribution of the two polymorphisms between patients and controls, and in every subgroup classified by the polymorphisms, both paraoxonase and arylesterase activities were lower in patients than in controls. This suggested that the enzyme activities of PON1 decreased in hemodialysis patients, independent of the genetic polymorphism [52]. In another series, 74 HD patients and 92 control subjects were studied. PON1 activity, PON1 genotype (55 and 192 PON1 allelic polymorphisms), and the lipid profile, including HDL subfractions, were reported [54]. HD patients had decreased median PON1 activity, lower mean HDL, as well as decreased mean HDL_3_ concentration. HDL retained about 70% of serum PON1 activity, almost completely carried (95%) by HDL_3_ [54]. Finally, PON1 activity was significantly lower in HD versus control subjects even after matching for the allelic polymorphism, which did not differ from control subjects. In another recent study, researchers measured PON activity in 377 hemodialysis patients using the substrates 4-nitrophenylacetate and phenylacetate and its variation over time [41]. The PON ratio was calculated from 4-nitrophenylacetate-derived activity divided by phenylacetate-derived activity. Frequency distribution of the PON1 ratio showed three different PON phenotypes. 74% of hemodialysis patients showed PON1 phenotype 1, 21% PON1 phenotype 2, and 5% PON1 phenotype 3. They observed a significant reduction of PON1 ratio with increasing dialysis time [41]. This suggests that qualitative changes in PON1 occur in a time-dependent manner, by which changes in one activity are higher than in others. Finally, a very recent study included 238 control subjects and 263 hemodialyzed patients [55]. Genotype frequencies were different between two compared groups only for L55M polymorphism, with control group having higher frequency of MM genotype. Again, they confirm that Q192R, L55M, and −108C-T polymorphisms are not causal factors leading to the lower PON1 activity in hemodialyzed patients [55]. Several other studies provide confirmatory results [38, 56–59].

## 6. Intervention Studies

### 6.1. Hemodialysis and Other Modalities Have Beneficial Effects on PON1 Activity

Hemodialysis is the most frequent modality of treatment for ESRD patients. To a degree that depends on the nature of the membrane employed, HD, although life-saving, is prooxidative and has the potential of negatively affecting PON1 activity. Our laboratory undertook a series of studies to address the effect of HD on PON1 activity. We enrolled 22 ESRD patients undergoing hemodialysis in whom paired pre- and postdialysis samples were studied along with 30 age-matched control subjects [46]. ESRD patients showed a 76% decrease in PON1 activity confirming previous reports. HD results in a significant, consistent increase in the activity of the antioxidant enzyme PON1, which ranged from 4 to 40% of the predialysis value. HDL-cholesterol, apoAI, free cholesterol (as a LCAT surrogate), HDL-subclasses, and TG did not change significantly after dialysis [46]. Changes in PON1 activity display a good correlation (*r* = 0.66, *P* < 0.001) with rates in which creatinine and urea are cleared suggesting that another cause for the observed lower PON1 concentrations in CRF is the retention of low-middle molecules (see below) and demonstrate a positive effect of hemodialysis in the delicate oxidant-antioxidant state of these patients, that should be weighed against other pro-oxidant effects that have also been shown to occur previously [46].

### 6.2. Effect of Membranes

In a recent study, the question whether HD membrane permeability has any influence on PON1 activity was addressed [60]. Forty-seven HD patients and 24 controls were enrolled. Blood samples were withdrawn after completion of 4-week treatment for a low-flux and a high-flux membrane. (TOS), total antioxidant status (TAS), and paraoxonase and arylesterase activities were measured in blood samples of the patients and the controls. Total oxidant status in HD patients (both types of membranes) were higher than controls while total antioxidant status and PON1 activity were lower in HD patients. There were no significant differences between “low-flux” and “high-flux” membranes in regard to oxidative stress parameters or PON1 enzyme activities. Membrane permeability seems to have no influence on oxidative stress parameters and PON1 enzyme activities [60].

### 6.3. HD and HDL Anti-Inflammatory Properties

By removing uremic toxins, dialysis may reduce LDL inflammatory and increase HDL anti-inflammatory properties. On the other hand, as stated above, exposure to dialyzer membrane tubing and impurities from dialysate could amplify LDL and HDL inflammatory activities. A recent study examined the effect of hemodialysis on LDL and HDL inflammatory activities [61]. ESRD patients had increased LDL chemotactic activity, reduced HDL anti-inflammatory activity, PON1 and glutathione peroxidase levels, and elevated plasma IL-6 before dialysis. Hemodialysis reduced LDL inflammatory and increased HDL anti-inflammatory activities. The beneficial effects of hemodialysis are in part mediated by heparin, which bears antioxidant lipolytic properties [61]. However, another study shows no difference in the ratio PON1/HDL in hemodialysis patients [62].

### 6.4. Vit C

 In a study with 42 ESRD in HD and 50 control subjects, CRF patients treated with vitamin C showed an increase of PON1 activity and a decrease of AGE and lipid hydroperoxides levels [63].

### 6.5. Quercetin and Cathechins

In a study with using the model of renal failure induced by ethylene glycol, it was reported that treatment impaired kidney composition, increased oxidative damage, and decreased serum paraoxonase and arylesterase activities [64]. Quercetin and cathechins enhanced antioxidant defenses—superoxide dismutase and PON1 activities, reducing oxidative damage suggesting that PON1 mediates the protective effects of flavonoids against kidney damage by oxidative stress [64].

### 6.6. EPO

 The effects of treatment of anemia with exogenous recombinant erythropoietin (EPO) beta and iron on levels of antibodies against oxidized low-density lipoproteins as well as on serum PON1 activity and concentration were studied in 49 predialysis patients with chronic renal disease [45]. After 6 months of treatment, compared with pretreatment values, the median levels of serum PON1 activity was slightly but significantly increased and the concentration of PON1 was significantly decreased. EPObeta and iron treatment of anemia of CRF promotes changes in serum PON1 activity and concentration that suggest a beneficial effect on oxidative stress.

## 7. Lactonase Activity of PON1 Is Lower in ESRD Patients and Is Restored by HD

As stated before, PON1catalyzes the hydrolysis of numerous substrates: lactones, thiolactones, esters, and phosphotriesters, including paraoxon, from which its misleading name is derived. Nevertheless, a consensus seems to be emerging that PON1 main physiological activity is acting as a lactonase [28, 65–72]. Findings reporting changes in the promiscuous esterase activities may not reflect changes in the physiological function. Indeed, the substrate docking moieties around the active site of PON1 differ for each major category of substrates, namely, the lactonase activity depends on a different region than the esterase activity [69, 73]. All of the studies reported above, including our own, had not employed lactones as substrates. In a study with 42 ESRD in HD and 49 control subjects, we found that our patients showed a significant 11% decrease in PON1 lactonase activity [74]. When we compared pre- and postdialysis samples, lactonase changed favorably as a result of dialysis, as did the other activities. This is worthy of note, given the likelihood that lactonase activity may be the most important physiological atheroprotective function of PON1. ESRD patients may be more susceptible to the harmful effects of lipid peroxidation than healthy subjects, lower serum lactonase activity would delay catabolism of oxidized phospholipids in LDL and oxidized macrophages, allowing more time for radical chain reactions to inactivate apolipoproteins and cell membrane proteins [74]. 

## 8. Mechanisms Proposed

### 8.1. Role of Uremic Toxins

#### 8.1.1. AGEs

We have shown that HD results in a significant, consistent increase in the activity of the antioxidant enzyme PON1. The effect correlates with the effectiveness of dialysis to clear creatinine and urea. This strongly suggests that elimination of some inhibiting low molecular factor may be responsible in part for the recovery of PON1 activity. Uremic toxins, therefore, may be putative mediators of PON1-deficient activity. Kidney failure patients have very high levels of advanced glycation endproducts; even more than diabetic patients [75–78]. AGE residues are formed by the action of carbonyls (glucose, methylglyoxal, and other dicarbonyls) on long- and short-lived cellular and extracellular proteins. Cellular proteolysis forms AGE-peptide and AGE-free adducts from these proteins, which are released into plasma for urinary excretion [75–78]. When we studied AGEs in our HD patients, the clearance of low-molecular-weight AGEs after hemodialysis explained 79% of the changes in PON1 activity and are hence a much better predictor than creatinine changes [46]. *In vitro* incubation of paraoxonase with serum ultrafiltrates showed a time- and concentration-dependent inhibition of PON1 by the ultrafiltrates, an inhibition that is up to 3 times higher (from 8 to 24%) when CRF patients are the source of the ultrafiltrate [46]. We showed that HD results in a significant, consistent increase in the activity of the antioxidant enzyme PON1. The effect correlates with the effectiveness of dialysis to clear creatinine and urea, and with the clearance of AGE adducts of low molecular weight. This effect was replicated *in vitro*, showing time and dose dependency. Our results suggest that another cause for the observed lower PON1 concentrations in CRF is the retention of low-middle molecules and demonstrate a positive effect of hemodialysis in the delicate oxidant-antioxidant state of these patients that should be weighed against other pro-oxidant effects that have also been shown to occur previously [46]. If the hypothesis that AGEs are the main culprits is proved in further research, this opens a putative therapeutic avenue for AGE blockers in ESRD.

#### 8.1.2. Acrolein

Renal failure patients share lower PON1 levels and high serum acrolein levels [79–81]. Acrolein is a highly reactive air pollutant of human health concern, chiefly as it is major component of cigarette smoke and also has several endogenous sources [79–81]. Acrolein oxidizes cysteine and forms adducts with lysine and histidine through the Maillard reaction, with deleterious consequences on protein function. Since PON1 has a cysteine residue critical for its antioxidant activity, which is moreover modulated by apoA-I, we hypothesized that acrolein could also have deleterious effects on PON1 activity [82]. We have shown that acrolein inhibits PON1 activity in HDL at micromolar concentrations and this inhibition is cancelled by n-acetylcysteine [82]. PON1 has 1 critical cysteine residue in its catalytic hydrophobic pocket. The results suggest that in conditions where local acrolein concentrations are elevated (atheroma plaque, sites of lipoperoxidation), acrolein-mediated loss of PON1 activity could be a plausible phenomenon. In a study with 40 ESRD in HD and 40 control subjects we found that our patients had a 3-fold increase in free acrolein when compared to control subjects. When we compared pre- and posthemodialysis samples, PON1 activity changed favorably as a result of dialysis, confirming our previous data [46, 82–84]. On average, free acrolein was 32% lower in postdialysis samples [82]. When we correlated the increments in PON1 activity resulting from the hemodialysis intervention, with the corresponding decrements in free acrolein we found a significant correlation. Our results suggest that high acrolein levels may in part be responsible for the low PON1 levels found in ESRD [82].

### 8.2. HDL Composition and PON1 Association/Dissociation

#### 8.2.1. HDL Composition

From the discussion above, it is clear that a certain consensus is emerging, favoring a multiprong explanation for PON1-deficient activity in CRF. Circulating inhibitors (uremic toxins and others) are likely candidates, together with decreased PON1 protein paired with qualitative changes in the molecule. Differences in allele distribution do not seem to play a role. PON1 activity largely depends on its association with apoAI and phospholipids in HDL, although a minor, free form can be found. In turn, HDL encompasses a wide range of particles with different sizes and diverse physiological functions. HDL subclasses are not homogeneous in PON1 content and activity. Small HDL_3_ contains most of this activity and carries most of the antioxidant capacity [13]. As CRF is associated with changes in HDL subclasses distribution and function, this may be another factor that compounds the observed decreased PON1 activity in these patients. However, reduced HDL levels *in vivo* may result in reduced HDL antioxidant capacity in these patients. In a study with 74 patients in HD, it was shown that mean HDL_3_ concentration is decreased [54]. Most of PON1 activity in HDL was carried (95%) by HDL_3_. The authors suggest that a key determinant of PON1 activity reduction in HD is the depressed HDL_3_ and not the genetic PON1 polymorphism [54].

#### 8.2.2. Free PON1 Is Not Higher in CRF Patients

Aviram's group has shown that free PON1 increases in diabetes patients and that glycation of HDL promotes dissociation of the enzyme from the particle [85–87]. They suggest that this mechanism may contribute to the lower activity found in these patients and to a dysfunctional more atherogenic HDL particle. We tackled the question whether increased free PON1 in CRF patients could be a reason for the lower activity [83]. Other than the classical routine findings in these patients, we confirmed that both PON1 triesterase and lactonase activities are reduced in ESRD patients on hemodialysis. The free triesterase activity is larger than the free lactonase activity in both populations [83]. Free PON1, however, was not significantly different between the groups and that was true for the activity against paraoxon and the more physiological lactonase activity [83].

## 9. Association with Other Biomarkers

### 9.1. Thiols

 PON1's only free sulfhydryl group is present at Cys284 and is associated with its activity even when it is not part of its active site; rather, it is part of a highly conserved stretch that includes active site histidine-285 [69, 73, 88–90]. Modification of the enzyme's free sulfhydryl group, such as via S-glutathionylation, leads to inactivation [91, 92]. In a study with 71 hemodialyzed patients and 87 healthy individuals, hemodialyzed patients had lower PON1 paraoxonase and arylesterase activity, concentrations of HDL, HDL_3_, and HDL_2_ and concentration of free thiol groups [55]. Decreased concentration of free thiol groups in sera suggest that the free thiol group (Cys284) in PON1 might also be oxidized, which can affect PON1 activity. In another study with 48 CRF patients on chronic maintenance hemodialysis, 41 patients on conservative management and 41 age-matched controls, serum PON1 activity correlated positively with protein thiols, and negatively with lipid hydroperoxides [93].

### 9.2. Ischemia-Modified Albumin (IMA)

The amino-terminal end of albumin binds transitional metals such as cobalt. When a specific motif (DAKK motif) in the N-terminus is damaged, this results in a reduced binding capacity for transitional metals [94–98]. This ischemia-modified albumin is formed in ischemic capillary beds as found in CVD, especially in the acute coronary syndromes, and has also been recently documented in patients with diabetes mellitus, hyperlipidemia, and metabolic syndrome [94–98]. Recently, prospective studies on CVD outcomes in ESRD using the new biomarkers of HDL dysfunction and/or oxidative stress, PON1 and IMA, have appeared in the literature [99–104]. In a pilot study, we explored this relationship in a small cohort of HD patients with ESRD [84]. We showed that PON1 levels were significantly and inversely correlated with IMA levels in these patients while such a clear correlation was not found in non-ESRD controls. An inverse correlation between PON1 and IMA levels indicate that low PON1 activity in these patients produces increased oxidative stress, leading to IMA formation. Monitoring serum PON1 and IMA simultaneously might thus provide another useful tool for the clinical pathologists as a prognostic biomarker of CVD in ESRD patients [84].

### 9.3. Cystatin C

 Serum cystatin C is an alternative, more specific marker of glomerular filtration rate [57]. In a study with 74 hemodialized, 171 renal transplanted patients, and 110 healthy controls, a negative correlation was found between PON1 activity and cystatin C level [43]. Homocysteine level correlated negatively with PON1 activity, and positively with cystatin C level. OxLDL and lipoperoxide levels were significantly higher in the renal patients. Cystatin C may be a good predictive factor for homocysteine levels but for the antioxidant status in patients with renal failure [42, 43].

### 9.4. Cortical Thickness

 In a study with 37 CRF patients and 18 controls, there was a positive correlation between renal cortical thickness and HDL levels and PON1 activity [105]. 

### 9.5. CRP

PON1 arylesterase activity and mass, C-reactive protein (CRP), cystatin C, were measured in 30 CRF patients and 30 control subjects [42]. PON1 activity and mass were inversely correlated with CRP in HD patients. The grouping of increased CRP and reduced PON1 may detect subjects at higher risk for cardiovascular complications [42, 106]. 

## 10. Predictive Value

Results from the multiple case-control studies reported in this paper provide substantial evidence that renal failure is associated with deficient PON1 activity and mass that are independent of changes in HDL-C. Evidence from prospective studies is more scarce but encouraging. One of these studies measured PON1 activity, concentration, and genetic polymorphisms with an interval of 6 years in 81 HD patients [51]. The relation between baseline cardiovascular risk factors and clinical events was investigated. During followup for 6 years, 42 deaths were recorded, including 24 fatal cardiovascular events. In univariate analyses, baseline PON1 concentration was associated with not only cardiovascular mortality, but also all-cause mortality during the period of follow-up, as were age, preexisting cardiovascular disease, and hemoglobin concentration [51]. In multivariate Cox regression analysis, PON1 concentration retained significant associations with cardiovascular mortality and all-cause mortality even after adjustment for other risk factors for CVD or mortality in HD patients. Significantly increased cardiovascular mortality and all-cause mortality were shown in the patients with low PON1 concentrations in Kaplan-Meier survival curves, suggesting that low PON1 concentration may be an independent predictor of cardiovascular mortality in maintenance HD patients [51]. In another study with 30 CRF patients undergoing HD, 30 patients with CRF under conservative treatment, and 30 healthy controls, basal PON1, salt-stimulated PON1 and arylesterase activities were lower in patients than controls [44]. Carotid intima-media thickness (IMT) was significantly higher in HD than in other CRF patients and both were significantly higher than controls. Linear regression showed that the most significant determinant of carotid IMT was PON1 arylesterase activity in HD. Modifying this risk factor could be salutatory in this patient population [44].

## 11. Conclusion

In addition to profound alterations in triglyceride, IDL- and HDL-cholesterol concentrations, classical hallmarks of renal failure, reduced serum paraoxonase activity has been clearly established in the past decade and could contribute to accelerated development of atherosclerosis in ESRD and in HD. PON1 activity and mass has been found consistently depressed in all studies papered. This is not due to associations of renal failure with specific phenotypes characterized by lower activity against paraoxon. PON1 lactonase and other activities all are the lower in ESRD patients. ESRD patients may be more susceptible to the harmful effects of lipid peroxidation than healthy subjects. Hemodialysis partially restores PON1 lactonase and the other activities. An association between PON1 activity recovery after dialysis has been found with creatinine changes, advanced glycation end products, and acrolein suggesting that uremic toxins may play a mechanistic role in PON1 inactivation. Lower PON1 activity in CRF patients is associated with low thiol concentration, high CRP, and is beneficially enhanced with vitamin C and flavonoids. Changes in HDL subclasses, namely, lower HDL_3_ in these patients may also play a role in PON1 lower activity. Some studies indicate that PON1 has an independent predictive value on CVD risk. These studies lay the ground for future studies around three axes: (1) mechanistic studies addressing the intimate explanation for low PON1 activity and mass; (2) prospective studies focusing on whether there is an added predictive value in measuring PON1 activity (and PON1 activity in HDL_3_) in this patient population; (3) intervention studies attempting to increase PON1 activity, studying outcomes providing information on effective delay in the progression of atherosclerosis.

## Abbreviations

AGE: Advanced glycation enedproducts
ApoAI: Apolipoprotein AI
CRF: Chronic renal failure
CAPD: Continuous ambulatory peritoneal dialysis
CRP: C-reactive protein
ESRD: End-stage renal disease
HD: Hemodialysis
HDL: High density lipoprotein
IDL: Intermediate density lipoprotein
IMT: Intima-media thickness.
